# Orthodontic Management of Different Stages and Grades of Periodontitis According to the 2017 Classification of Periodontal Diseases

**DOI:** 10.3390/dj13020059

**Published:** 2025-01-29

**Authors:** Nada Tawfig Hashim, Shahistha Parveen Dasnadi, Hassan Ziada, Muhammed Mustahsen Rahman, Ayman Ahmed, Riham Mohammed, Md Sofiqul Islam, Rohan Mascarenhas, Bakri Gobara Gismalla, Neamat Hassan Abubakr

**Affiliations:** 1 Periodontics Department, RAK College of Dental Sciences, RAK Medical & Health Sciences University, Ras-AlKhaimah 12973, United Arab Emirates; mustahsen@rakmhsu.ac.ae; 2 Orthodontics Department, RAK College of Dental Sciences, RAK Medical & Health Sciences University, Ras-AlKhaimah 12973, United Arab Emirates; shahistha.parveen@rakmhsu.ac.ae; 3 Clinical Science Department, School of Dental Medicine, University of Nevada, Las Vegas, NV 89106, USA; hassan.ziada@unlv.edu; 4 Periodontics Department, College of Dentistry, Nile University, Khartoum 1847, Sudan; ayman.ahmed@nileuniversity.edu.sd; 5 Oral Surgery Department, RAK College of Dental Sciences, RAK Medical & Health Sciences University, Ras-AlKhaimah 12973, United Arab Emirates; riham.abdelraouf@rakmhsu.ac.ae; 6 Operative Department, RAK College of Dental Sciences, RAK Medical & Health Sciences University, Ras-AlKhaimah 12937, United Arab Emirates; sofiqul.islam@rakmhsu.ac.ae; 7 Orthodontics Department, Yenepoya Dental College, Mangaluru 575018, India; rohanmasc@yenepoya.edu.in; 8 Oral Rehabilitation Department, Faculty of Dentistry, University of Khartoum, Khartoum 11115, Sudan; bakri.gobara@uofk.edu; 9 Biomedical Science Department, School of Dental Medicine, University of Nevada, Las Vegas, NV 89106, USA; neamat.hassan@unlv.edu

**Keywords:** orthodontic treatment, 2017 Periodontal Classification, Stage III and IV periodontitis, interdisciplinary management, bone regeneration, guided tissue regeneration

## Abstract

**Background/Objectives:** The 2017 Periodontal Classification offers a comprehensive framework for the diagnosis and management of periodontitis based on staging and grading criteria. Orthodontic therapy is increasingly incorporated into the management of periodontitis to rectify malocclusion, pathological tooth migration, and occlusal stability. Nonetheless, few data directly correspond with this revised classification scheme. The objective of this systematic review is to figure out the influence of orthodontic therapy on periodontal outcomes in patients with Stage III and IV periodontitis, as categorized by the 2017 framework. **Methods**: A systematic review was performed in accordance with the PRISMA 2020 principles. The databases examined were PubMed, Web of Science, Scopus, and Google Scholar. The evaluation focuses on research published from 2012 to 2024. Seventeen studies were assessed after the application of the inclusion criteria. Key outcomes included clinical attachment level (CAL) improvement, probing depth (PD) decrease, and radiographic bone fill. **Results**: The integration of orthodontic treatment with periodontal therapy markedly enhanced CAL (mean gain: 4.35–5.96 mm), decreased PD (mean reduction: 3.1–6.3 mm), and facilitated radiographic bone regeneration (mean vertical fill: 4.89 mm). Patients with Stage IV Grade C periodontitis had the most significant improvement, especially with early orthodontic intervention subsequent to regenerative treatment. Prolonged follow-ups (up to 10 years) validated consistent results. **Conclusions**: Orthodontic intervention, as a supplementary measure to periodontal therapy, improves results in severe periodontitis, especially in Stage III and IV patients. These results underscore the need for multidisciplinary teamwork and defined protocols for including orthodontics in periodontitis therapy.

## 1. Introduction

Periodontal disease is a significant worldwide health problem and a main cause of tooth loss, characterized by progressive inflammation and destruction of tooth-supporting structures [1]. Beyond the oral cavity, the systemic implications of periodontitis, such as its link to cardiovascular disease and diabetes, highlight the need for appropriate treatment approaches [2]. Furthermore, severe cases of periodontitis cause considerable functional limitations, such as pathological tooth migration and occlusal discrepancies, impairing patients’ quality of life [3]. Orthodontic treatment has recently emerged as a viable adjunct to periodontal therapy. Orthodontics not only restores functional and cosmetic harmony by correcting malocclusions and dispersing occlusal stresses, but it also stabilizes long-term periodontal results [4]. Despite its promise, the integration of orthodontics into periodontitis management remains inconsistent, owing to a lack of defined standards and evidence-based protocols. This gap is particularly crucial in situations with advanced periodontitis when complete multifaceted approaches are required for optimal outcomes [5].

The introduction of the 2017 Classification of Periodontal and Peri-Implant Diseases and Conditions marked a significant shift in the diagnostic framework for periodontitis. This new system categorizes periodontitis based on staging (severity and complexity of management) and grading (rate of progression and risk factors such as smoking and diabetes), allowing for a more comprehensive understanding of disease presentation and progression [6]. While this classification has provided clarity in diagnosis and treatment planning, there remains a scarcity of studies specifically designed to align with this updated classification, particularly in the context of orthodontic management of periodontitis patients [7].

The integration of orthodontic and periodontal therapies within the framework of the 2017 classification highlights a novel interdisciplinary approach to managing advanced periodontitis [8].

Unlike previous classifications, the 2017 system facilitates a structured understanding of disease complexity and progression, allowing for better synergy between disciplines [9,10]. The novelty of this approach lies in its capacity to address both the systemic and localized factors contributing to periodontal disease progression [6]. By incorporating risk factors such as smoking and diabetes into the grading system, the 2017 classification provides a basis for more comprehensive treatment planning [6]. For example, tailored orthodontic forces can minimize the risk of further periodontal damage, while regenerative therapies can simultaneously address underlying bone and attachment loss. This dual focus on alignment and regeneration distinguishes the interdisciplinary approach as a cornerstone of modern periodontal care [11].

Moreover, this approach emphasizes the importance of collaboration among specialists. Orthodontists and periodontists working within the 2017 classification framework can better coordinate their efforts to achieve long-term stability and improved patient outcomes [5].

The absence of well-defined guidelines or substantial clinical evidence addressing orthodontic treatment across the various stages and grades of periodontitis presents a critical gap in the literature. As a result, clinicians often rely on extrapolating data from studies conducted under the previous classification systems [9,10], leaving uncertainties regarding the appropriateness and efficacy of orthodontic intervention in patients categorized under the Stage III and IV Grade C framework [6]. Therefore, this systematic review attempts to address this gap by evaluating studies that either explicitly conform to the 2017 classification or maybe retrospectively categorized based on their reported data. The review also aims to clarify the practicality, results, and obstacles of integrating orthodontic treatment in the management of advanced periodontitis, especially in instances when orthodontic intervention is crucial for rectifying secondary malocclusions and occlusal stress. The findings of the study may provide a basis for future interdisciplinary recommendations and underscore the need for tailored orthodontic strategies in the treatment of periodontitis throughout its various stages and grades.

## 2. Methodology

### 2.1. Focused Question (PICO Framework)

In patients with periodontitis of varying stages and grades (Population), how does orthodontic treatment (Intervention) influence periodontal health and orthodontic treatment outcomes (Outcome) when compared across different stages and grades of periodontitis (Comparison)?

### 2.2. Search Strategy

This systematic review was registered in the International Prospective Register of Systematic Reviews (PROSPERO) under the registration number CRD42025630425, ensuring methodological transparency and adherence to established guidelines. The protocol for this review will be publicly accessible through the database after complete registration. The review followed the Preferred Reporting Items for Systematic Reviews and Meta-Analyses (PRISMA 2020) guidelines [12].

A comprehensive electronic literature search was conducted across MEDLINE (PubMed), Web of Science, Scopus, and Google Scholar, covering the period from 1 January 2017 to 14 December 2024. This time frame was selected to focus on studies published after the introduction of the 2017 Classification of Periodontal and Peri-Implant Diseases and Conditions, ensuring that the included research aligns with the updated staging and grading framework. However, studies published before 2017 were also considered for inclusion if they contained cases of severe periodontitis, aggressive periodontitis, or chronic periodontitis with well-documented attachment loss, bone destruction, or radiographic evidence that allowed for retrospective classification into Stages III or IV and Grades B or C according to the 2017 classification [6]. This approach is supported by Raza et al. (2024), who demonstrated the feasibility of accurately reassigning diagnoses using the 2017 framework. Their methodology of leveraging radiographic bone loss (RBL), probing pocket depth (PPD), and clinical attachment loss (CAL) demonstrates that available clinical parameters can reliably support reclassification into stages and grades [13]. In this systematic review, similar methods were employed to ensure consistency in categorizing cases where retrospective data was sufficient. Aggressive periodontitis cases were predominantly reclassified as Stage III or IV, Grade C, and chronic periodontitis cases as Stages II or III, Grades B or C. Subgroup analyses further confirmed that the variability introduced by reclassification had minimal impact on key outcomes, reinforcing the robustness of the 2017 classification for standardizing periodontitis diagnosis and treatment.

The search strategy for this systematic review was developed using MeSH terms and keywords to ensure a comprehensive retrieval of relevant studies across multiple databases. For PubMed, the search terms were determined with the support of the MeSH Browser Tool and included Periodontitis [Mesh] AND (Orthodontics [Mesh] OR Orthodontic Treatment [Mesh] OR Periodontal Disease Classification 2017 [Mesh]) AND (Staging [Mesh] OR Grading [Mesh]). In SCOPUS, the query was formulated as KEY (periodontitis) OR KEY (periodontal AND disease) OR KEY (periodontal AND therapy) AND KEY (orthodontics) OR KEY (orthodontic AND treatment) OR KEY (periodontal AND disease AND classification) AND KEY (staging) OR KEY (grading). Similarly, for Web of Science, the search query was structured as AK = (Periodontitis OR Periodontal AND disease OR Periodontal AND therapy) AND AK = (Orthodontics OR Orthodontic AND treatment OR Periodontal AND disease AND classification) AND AK = (Staging OR Grading). The keywords used for the Google Scholar database search were “Periodontitis” OR “Periodontal disease” OR “Periodontal therapy” AND “Orthodontics” OR “Orthodontic treatment” OR “Periodontal disease classification 2017” AND “Staging” OR “Grading.” The search was conducted using Publish or Perish software, with a limitation set to retrieve the 500 most relevant results. This approach ensured a focused and comprehensive identification of studies related to orthodontic treatment in patients with periodontitis classified by the 2017 periodontal disease classification system (Table 1).

The authors used a “snowball” search strategy to identify additional relevant studies. This involved reviewing the reference lists of articles selected for full-text review and utilizing Google Scholar to locate studies that cited these articles. The search was restricted to publications in English. To ensure inclusivity and reduce potential bias, the authors did not restrict the search to specific study designs, acknowledging that valuable data could be obtained from a variety of research methodologies. N.T.H. and S.P.D. independently conducted the database searches using the same predefined terms. All authors collaborated to evaluate whether the identified studies fulfilled the inclusion criteria. Finally, N.T.H. and S.P.D. jointly reviewed the included studies to extract the required data systematically.

### 2.3. Selection of Studies

This systematic review aimed to evaluate the orthodontic management of patients with periodontitis using the 2017 periodontal disease classification system. The review aimed to determine how different stages and grades of periodontitis influence the outcomes of orthodontic treatment and how these outcomes compare across the staging and grading categories. The hypothesis was that orthodontic treatment, when tailored to the stage and grade of periodontitis, would improve periodontal health and orthodontic stability.

The eligibility criteria for this systematic review were meticulously established to guarantee the inclusion of articles that align with the study aims. Studies were considered if they included adult patients diagnosed with periodontitis, as categorized by the 2017 Periodontal Disease Classification system, and reported quantifiable clinical results associated with orthodontic therapy. The results included measures including CAL improvement, PD decrease, and radiographic bone restoration. Only research using multidisciplinary methodologies between orthodontists and periodontists was included. The review was restricted to research published in peer-reviewed publications, accessible in full text, and composed in English to ensure consistency and quality. Furthermore, studies completed before 2017 were included if their clinical and radiographic data permitted retroactive categorization according to the 2017 staging and grading criteria. This guaranteed that significant data from previous research may still be included in the evaluation, contingent upon their adherence to the revised diagnostic criteria. Exclusion criteria were established to exclude research that was either irrelevant or methodologically unsound. The selected papers were case reports, case series, narrative reviews, systematic reviews, meta-analyses, animal studies, and research involving pediatric populations (patients under 18 years of age). Additionally, studies without quantifiable orthodontic or periodontal results or those with inadequate clinical information for retrospective categorization according to the 2017 framework were excluded (Table 2).

### 2.4. Risk of Bias in Individual Studies

In the initial stage of study selection, each reviewer independently evaluated the titles and abstracts to reduce potential bias. The level of agreement between reviewers was assessed using Cohen’s κ test, ensuring a systematic approach to the selection process [14]. Any disagreements regarding the inclusion or exclusion of a study were resolved through thorough discussions among the authors until a consensus was achieved. This approach helped ensure objectivity and reliability when selecting studies for review.

### 2.5. Sample Selection

The sample selection for this review was conducted independently by two reviewers (N.T.H. and S.P.D.) to ensure a systematic and unbiased approach. The evaluation of each study’s procedural quality was based on specific criteria, including clear inclusion and exclusion criteria, adequate representation of participants across different stages and grades of periodontitis, and sufficient detail about the methodology used to assess orthodontic treatment outcomes. Studies were included if they addressed the interaction between orthodontic treatment and periodontitis progression using the 2017 periodontal classification system. Additionally, studies conducted before 2017 were considered if they provided detailed clinical data (such as the extent of attachment loss, bone destruction, and clinical/radiographic records) that allowed for retrospective staging and grading in alignment with the updated classification. This ensures the inclusion of studies on severe or aggressive periodontitis cases, chronic periodontitis with advanced attachment loss, or bone destruction, facilitating proper categorization according to the new system. The reviewers also ensured that the studies clearly defined sample characteristics and a reliable methodology for assessing periodontal and orthodontic outcomes.

### 2.6. Risk of Bias in Sample Selection

The risk of bias evaluation was carried out to determine the methodological rigor of the included research based on their design. The Cochrane Risk of Bias 2 (ROB 2) method was used to review randomized controlled trials (RCTs) [14], which looked at areas such as randomization, variations from planned treatments, missing outcome data, outcome assessment, and selective reporting. Retrospective cohort studies were assessed using the ROBINS-I technique, which focused on confounding factors, selection bias, intervention categorization, missing data, and outcome measurement. The Newcastle-Ottawa Scale was used to evaluate case-control studies, with a focus on participant selection, group comparability, and measuring exposure and outcomes. Cross-sectional studies were evaluated using the Joanna Briggs Institute (JBI) checklist, which included questions on participant selection, measurement validity, managing missing data, and reporting bias [15].

### 2.7. Data Extraction

Following a consensus on the selection of articles, data extraction was carried out by the two reviewers. The information extracted from each study included the following:

- Citation details (first author and publication year)
- Type of study design
- Description of the intervention and control groups
- Diagnosis criteria or classification used
- Clinical parameters assessed
- Reported outcomes
- Length of follow-up
- Number of participants
- Whether a sample size calculation was performed
- Age range and standard deviation of participants
- Gender distribution within the sample

## 3. Results

### 3.1. Study Selection

**Figure 1:**
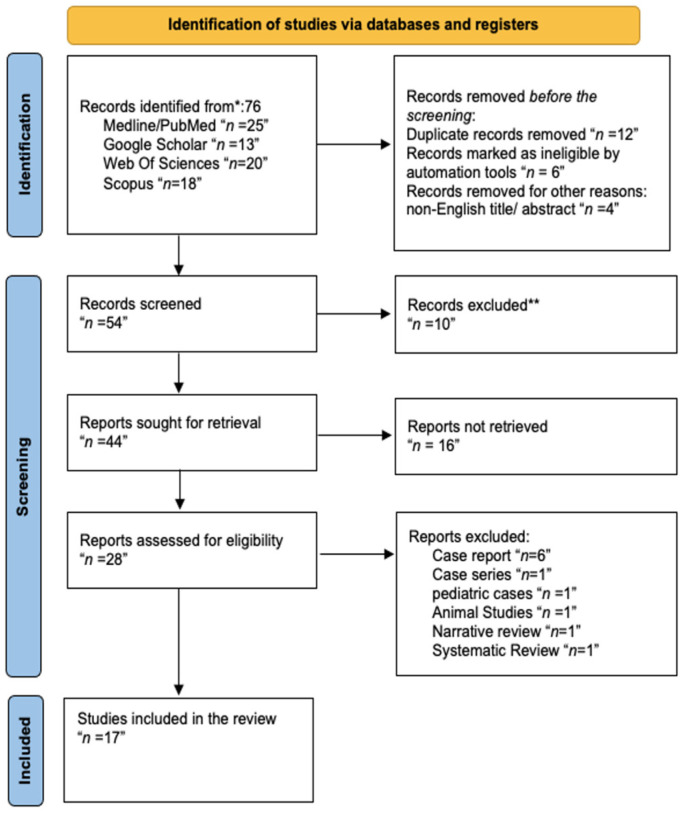
Screening flowchart for the investigated studies following the PRISMA guidelines. ** The step was performed by humans with no automation tools.

Figure 1 details the study selection process based on the PRISMA statement [12].

A comprehensive database search using the keywords “Orthodontics”, “Periodontology”, “staging”, “grading”, “periodontal disease classification”, “aggressive periodontitis”, “interdisciplinary collaboration”, and “treatment outcomes” identified 76 articles. These studies were distributed as follows: 25 from PubMed, 18 from Scopus, 20 from Web of Science, and 13 from Google Scholar.

After removing duplicates, 54 articles were screened by title and abstract. Of these, 28 articles underwent a full-text review to ensure relevance and adherence to the eligibility criteria. Following this detailed assessment, 20 studies were included in the systematic review. These selected articles primarily focused on the orthodontic management of patients with periodontitis, which was classified under the 2017 Periodontitis Classification, including those categorized as Stage III or IV, Grade C. Additionally, studies conducted before 2017 were included if sufficient clinical data (such as bone loss, attachment level, or extent of disease) allowed for retrospective staging and grading based on the updated classification system. The included studies span publication from 2010 to 2023 and encompass diverse methodologies, clinical settings, and interdisciplinary approaches to optimize treatment outcomes.

A total of 11 out of the 28 studies initially identified for this systematic review were excluded based on specific criteria [16,17,18,19,20,21,22,23,24,25,26]. One study was excluded for being a pediatric case [20], as the review targeted adult populations. Additionally, six case reports [16,17,18,19,23,24], one case series [25], one narrative review, and one systematic review were excluded due to their limited generalizability and lack of robust data applicable to broader clinical practice [22,26]. One animal study was excluded as its findings could not be directly extrapolated to human subjects [21]. The remaining 17 studies met the inclusion criteria and were subjected to full-text analysis for data extraction and synthesis (Table 3).

Risk of Bias Assessment

Following the qualitative synthesis of the included studies, the risk of bias assessment was conducted to evaluate the methodological quality of the evidence. Each study was assessed based on its design, using standardized tools such as the ROBINS-I, Newcastle-Ottawa Scale, JBI Checklis**t**, as well as the Cochrane Risk of Bias 2 (ROB 2) for randomized controlled trials [15]. The detailed assessment is summarized in Table 4.

The results of the risk of bias evaluation are as follows:

- Retrospective Cohort Studies: Moderate to serious risk of bias was observed, primarily due to issues with confounding, selection bias, and incomplete data reporting. These limitations reflect the inherent constraints of retrospective designs.
- Cross-Sectional Studies: Moderate risk of bias was noted, largely attributed to challenges in participant selection and external validity. Measurement validity and reporting quality were generally acceptable.
- Controlled Clinical and Pilot Studies: Moderate risk of bias arose from small sample sizes, variability in intervention protocols, and incomplete follow-up.
- Randomized Controlled Trials (RCTs): Low to moderate risk of bias was found. Most RCTs demonstrated robust randomization and blinding procedures but were limited by occasional selective reporting and incomplete data.

**Figure 2:**
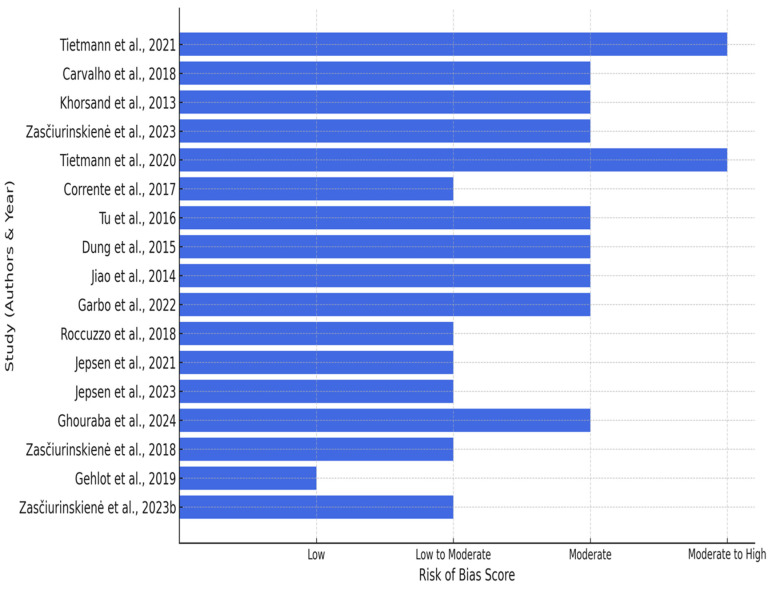
Risk of bias across included studies [28,29,30,31,32,33,34,35,36,37,38,39,40,41,43,44].

RCTs exhibited a low to moderate risk of bias, with notable concerns regarding deviations from intended treatments and inadequate reporting of outcomes. Retrospective cohort studies are associated with significant bias risks due to challenges in addressing confounding factors and handling missing data. Cross-sectional studies demonstrated a significant risk of bias, especially regarding participant selection and outcome assessment (Figure 2).

### 3.2. General Characteristics of the Included Studies

The general characteristics of the 17 included studies are summarized in the table, detailing study populations, sample sizes, and demographic data. Most studies investigated patients with advanced periodontal conditions, particularly Stage IV, Grade C periodontitis, reflecting significant bone loss and attachment levels [27,28,30,31,35]. Sample sizes ranged from 8 to 121 patients aged 18 to 78 years. Gender distribution was provided in 15 studies, while one study reported only the percentage of female participants, and one did not specify sex distribution. Most studies aligned with the 2017 periodontal classification system, while older studies were retrospectively staged and graded based on available clinical and radiographic data (Table 5).

### 3.3. Detailed Characteristics of the Studies

The studies included in this review evaluated the impact of orthodontic treatment of periodontal clinical parameters as part of comprehensive periodontal therapy. The patient populations exhibited varying degrees of periodontitis, ranging from mild to severe cases, classified according to the 2017 Periodontal Classification System. Orthodontic interventions across these studies involved fixed appliances, clear aligners, and intrusion techniques**,** often combined with regenerative procedures like guided tissue regeneration (GTR) or bone grafting. (Table 6).

### 3.4. Main Outcomes of the Studies Based on the 2017 Periodontal Classification

#### 3.4.1. Orthodontic and Periodontal Interventions

- Combination of GTR and Orthodontic Intrusion:

Ghouraba et al. (2024) compared the outcomes of GTR followed by orthodontic intrusion (OI) versus OI followed by GTR in treating over erupted teeth with angular bone loss. The study involved 10 patients and found that the group receiving GTR first demonstrated significant short-term improvement. In contrast, the OI-first group exhibited better long-term stability with reduced PD and tooth migration at one-year follow-up [27].

- Orthodontic Treatment for Severe Periodontitis:

Jiao et al. (2019) evaluated the effect of orthodontic treatment on patients with Stage IV, Grade C periodontitis. Among 24 participants, no significant changes were observed in PD or bleeding on probing (BOP), suggesting that periodontal stability can be maintained following orthodontic treatment [28].

- Combined Periodontic and Orthodontic Therapy:

Corrente et al. (2003) assessed the effects of periodontal surgery and orthodontic intrusion in patients with advanced periodontal disease and extruded incisors. The study reported significant reductions in PD (average 4.35 mm) and CAL gains (mean 5.50 mm**)** after 10 months, indicating positive outcomes without adverse effects such as root resorption [29].

- Interest in Orthodontic Treatment in Periodontitis Patients:

Zasčiurinskienė et al. (2023) investigated the interest in orthodontic treatment among 96 adults with Stage III–IV periodontitis. The study found that 56.3% of participants expressed interest, with Stage IV, Grade C periodontitis predicting higher orthodontic treatment needs [30].

#### 3.4.2. Long-Term Stability and Follow-Up

- Long-term Evaluation of Regenerative-Orthodontic Therapy:

Tietmann et al. (2023) conducted a 10-year study on 22 patients with Stage IV periodontitis, assessing the effectiveness of regenerative treatment combined with orthodontics. The study reported 4.48 mm radiographic bone level (RBL) gains, 90% pocket closure, and minimal tooth loss (4.5%), demonstrating the long-term benefits of combining orthodontic and regenerative treatments [31].

- Timing of Orthodontic Treatment and Periodontal Healing:

Zasčiurinskienė et al. (2018) explored the effect of orthodontic treatment timing in 50 patients. The control group, which received periodontal therapy before orthodontics, exhibited more healed PD sites (4–6 mm) than the test group. The six-year follow-up underscores the importance of timing in achieving favorable periodontal outcomes [32].

#### 3.4.3. Patient Outcomes and Quality of Life

- Orthodontics and Quality of Life Improvements:

Jepsen et al. (2023) conducted a 24-month study involving 43 patients, comparing early (4 weeks post-regenerative surgery) versus late (6 months) orthodontic treatment. The early treatment group achieved a significantly higher CAL gain (5.96 mm) and better pocket closure compared to the late group. Both groups reported improved oral health-related quality of life (GOHAI) [36].

- Fixed Orthodontics in Periodontally Compromised Patients:

Gehlot et al. (2022) studied 36 patients undergoing fixed orthodontic treatment. The study demonstrated significant improvements in periodontal health across CAL, PD, and plaque indices, with no differences between test and control groups, supporting the safety and efficacy of fixed orthodontic appliances in periodontitis patients [33].

- Aggressive Periodontitis and Orthodontics:

Carvalho et al. (2018) evaluated orthodontic effects in patients with aggressive periodontitis (AP). The study, which involved 20 participants, reported significant improvements in clinical parameters, including PPD reduction (−0.29 mm) and CAL gain (+0.38 mm) [34].

#### 3.4.4. Radiographic Bone Gains and Intrusion Techniques

- Radiographic and Clinical Improvements:

Dung et al. (2024) examined the long-term impact of immediate orthodontic treatment after regenerative procedures in 9 patients with 17 intrabony defects. The study reported an average PD reduction of 3.94 mm and CAL gain of 3.47 mm over 12.8 years [40].

- Intrusion Techniques for Over-erupted Teeth:

Khorsand et al. (2013) evaluated orthodontic intrusion in eight patients with aggressive periodontitis, demonstrating significant reductions in PD and defect size, contributing to improved periodontal health over six months [38].

## 4. Discussion

This systematic review sought to evaluate the impact of orthodontic treatment combined with periodontal therapy on clinical, radiographic, and patient-reported outcomes in periodontitis patients under the 2017 Periodontal Classification framework. While the findings affirm the significant potential of interdisciplinary approaches, critical evaluation highlights limitations in the current evidence base, variability in treatment protocols, and unresolved controversies.

The adoption of the 2017 Periodontal Classification represents a pivotal shift in understanding and managing periodontitis, providing a standardized framework that incorporates staging and grading to assess disease severity and progression [44]. Evidence utilizing this updated classification allows for a more nuanced evaluation of treatment outcomes, enabling clinicians to align interventions with specific disease profiles [45].

For instance, the classification of periodontitis into Stages III and IV, Grade C, as shown in studies such as that carried out by Jepsen et al. (2023), emphasizes the need to adjust orthodontic and periodontal treatments to advanced cases that exhibit severe attachment loss and rapid progression [36]. Before the 2017 update, the absence of such stratification often resulted in uneven comparisons across trials and clinical uncertainty in treatment recommendations. This change in classification helps to create customized treatment plans and improves the capacity to assess treatment efficacy [46].

The clinical implications of adopting the 2017 classification are substantial [46]. It emphasizes the need for clinicians to integrate disease staging and grading into their diagnostic and therapeutic decision-making processes [46]. For instance, managing patients with Stage IV periodontitis necessitates a comprehensive approach, addressing both the functional and esthetic impacts of pathologic tooth migration alongside regenerative and orthodontic interventions [26].

Studies adopting the new classification have underscored the importance of coordinating orthodontic alignment with guided tissue regeneration (GTR) or bone grafting to maximize CAL gain and PD reduction [40,41,42]. These findings suggest that clinicians should prioritize interdisciplinary collaboration and consider patient-specific factors, such as disease severity and systemic conditions, to optimize outcomes.

The new classification also provides guidance on practical aspects of patient management [46]. Clinicians should incorporate detailed risk assessments, including systemic factors such as diabetes or smoking, to tailor interventions effectively [46]. For example, Grade C cases with rapid progression may require more intensive monitoring and adjunctive therapies, such as host modulation, alongside standard periodontal and orthodontic care. Additionally, treatment planning should account for the impact of orthodontic forces on compromised periodontal tissues, necessitating the use of controlled forces to minimize risks of further attachment loss [47]. By considering these aspects, clinicians can enhance treatment predictability and long-term success.

In the present systematic review, the prevalence of observational and retrospective research highlights a deficiency in robust evidence, particularly the absence of randomized controlled trials (RCTs). Although observational studies provide significant insights into real-world clinical situations, their intrinsic constraints, such as possible bias and absence of standardization, weaken the robustness of results [48]. To mitigate this, we performed a thorough risk-of-bias evaluation, assuring prudent interpretation of findings from trials with elevated bias risk.

A critical analysis of the findings reveals substantial variability in treatment protocols, particularly concerning the sequencing of orthodontic and regenerative therapies. For example, studies like Ghouraba et al. (2024) demonstrate that guided tissue regeneration (GTR) followed by orthodontic intrusion yields better short-term defect correction, whereas orthodontic intrusion followed by GTR provides superior long-term outcomes [27]. This highlights the importance of tailoring treatment plans to individual clinical scenarios. Similarly, the timing of orthodontic intervention post-regeneration remains a point of contention. Studies such as Jepsen et al. (2021) suggest that early orthodontic correction accelerates bone fill and clinical attachment level (CAL) gains, while others advocate for delayed intervention to allow for complete regenerative healing [37]. These divergent findings underscore the need for further comparative studies to determine optimal treatment strategies.

The choice of orthodontic modality also significantly influences outcomes. Fixed appliances have been effective in managing severe malocclusion and pathologic tooth migration (PTM), yet they pose risks of increased plaque accumulation and potential periodontal damage [30,43]. Conversely, clear aligners, while offering esthetic and minimally invasive alternatives, lack robust evidence supporting their efficacy in periodontally compromised patients [33]. The paucity of direct comparative studies between these modalities limits definitive recommendations, reinforcing the need for focused research to delineate their relative benefits and risks.

Evidence quality also varies across measured outcomes. High-quality evidence supports significant improvements in CAL, particularly in advanced periodontitis cases (Stages III and IV, Grade C), as shown by Jepsen et al. (2023) and Tietmann et al. (2023). However, evidence for probing depth (PD) reduction and radiographic bone fill remains moderate, with inconsistencies attributable to differences in defect morphology, regenerative techniques, and follow-up durations. Extended observation periods, such as the 10-year follow-ups reported by Roccuzzo et al. (2018), are essential to evaluate the sustainability of outcomes and the risk of relapse [36,41,42].

The variability in outcomes associated with regenerative techniques is also an issue of controversy. Although research shows substantial vertical and horizontal bone fill, inconsistent results imply that procedure selection and defect shape have a crucial role in outcome. Intrabony defects demonstrate a more consistent benefit from GTR compared to horizontal bone loss, indicating the need for personalized therapeutic strategies. While short-term results for bone regeneration are promising, the long-term durability of these outcomes is still uncertain [27,38,41]. Future research should concentrate on identifying factors that promote sustained bone health and reduce the risks of relapse

Patient-reported outcomes (PROs) represent a significant gap in the literature. Despite the clear clinical benefits, limited attention has been given to assessing quality of life, satisfaction, and psychosocial impacts of treatment. While Jepsen et al. (2023) reported improvements in oral health-related quality of life, such findings are not consistently represented across studies [36]. Incorporating validated PRO measures in future research will provide a more comprehensive understanding of the broader benefits of orthodontic-periodontal interventions.

Potential reporting bias further complicates interpretation, as studies with positive outcomes are more likely to be published, potentially underrepresenting unfavorable or inconclusive results. Our review employed a comprehensive search strategy and critically assessed included studies for bias, yet addressing this limitation requires greater transparency in publishing all research outcomes.

While this review highlights the promising outcomes of combining orthodontic treatment with periodontal therapy, it also identifies critical gaps and areas of controversy. The variability in treatment protocols, lack of long-term data, and limited focus on PROs underscore the need for more rigorous and comprehensive research. Future studies should prioritize RCTs, standardized protocols, and extended follow-ups to refine therapeutic strategies and improve patient-centered care. By addressing these limitations, the evidence base can better support the integration of orthodontics and periodontics into clinical practice, ultimately enhancing outcomes for patients with periodontitis.

## 5. Conclusions

This systematic review emphasizes the enormous advantages of combining orthodontic treatment with periodontal therapy, especially in advanced periodontitis patients defined by the 2017 Periodontal Classification. Better therapeutic results, such as CAL gain and PD reduction, were consistently seen when multidisciplinary teamwork and tailored treatment planning were prioritized. However, the absence of randomized controlled trials and defined methodologies emphasizes the need for more research to improve evidence-based clinical practice. Future research should concentrate on long-term follow-up studies and PROs in order to provide comprehensive guidlines for treating periodontally compromised individuals.

## Figures and Tables

**Table 1:** Search strategy for systematic review.

Database	Search Terms/Keywords	Filters Applied	Results Retrieved
PubMed	(“Periodontitis”[MeSH] AND (“Orthodontics”[MeSH] OR “Orthodontic Treatment”[MeSH]) AND “Periodontal Disease Classification 2017”[MeSH]) AND (Staging[MeSH] OR Grading[MeSH])	English language, 2017–2024, Full-text	25
Scopus	KEY(“Periodontitis” OR “Periodontal Disease” AND “Orthodontics” OR “Orthodontic Treatment” AND “Periodontal Disease Classification 2017”) AND (“Staging” OR “Grading”)	English language, Peer-reviewed journals	18
Web of Science	AK = (“Periodontitis” OR “Periodontal Therapy” AND “Orthodontics” OR “Orthodontic Treatment” AND “Periodontal Disease Classification 2017” AND “Staging” OR “Grading”)	English language, 2017–2024, Peer-reviewed	20
Google Scholar	“Periodontitis” OR “Periodontal Disease” AND “Orthodontics” OR “Orthodontic Treatment” OR “Periodontal Disease Classification 2017” AND “Staging” OR “Grading”	Top 500 results, English language	13

**Table 2:** Selection criteria for studies included in the systematic review.

Inclusion Criteria	Exclusion Criteria
Full-text availability	Case reports/case series
English language	Systematic reviews
Adult patients with periodontitis	Meta-analyses
Studies focusing on orthodontic treatment tailored to staging and grading	Historic reviews
Reported clinical outcomes specific to periodontal health and orthodontics	Conference papers
Studies utilizing the 2017 classification system for periodontitis	Letters to the editor
Interdisciplinary approaches between orthodontists and periodontists	Animal studies
Published in peer-reviewed journals	Patients younger than 11 years old
Minimum follow-up period for assessing outcomes	Studies without measurable orthodontic or periodontal outcomes
Focused on the interaction between orthodontic and periodontal care	
Studies published before 2017 were also included if they provided sufficient clinical details (e.g., severity of attachment loss and bone destruction) allowing for retrospective staging and grading under the 2017 classification.	

**Table 3:** List of excluded studies and reasons for exclusion.

Study Title	Reason for Exclusion	Reference
A Case of Orthodontics Treatment for Generalized Aggressive Periodontitis	Case Report	[16]
Digital Orthodontic Setup and Clear Aligners System for Treating Adult Patients with Periodontitis	Case Report	[17]
Interdisciplinary Therapy for Severe Periodontitis with Angle Class II Division 1 Malocclusion	Case Report	[18]
Aggressive Periodontitis with a History of Orthodontic Treatment	Case Report	[19]
One Year Follow-Up of a 4-Year-Old Caucasian Girl Diagnosed with Stage IV Grade C Localized Periodontitis	Pediatric Case	[20]
Periodontal Response to Orthodontic Tooth Movement in Diabetes-Induced Rats	Animal Study	[21]
Orthodontic Tooth Movement After Periodontal Regeneration of Intrabony Defects	Narrative Review	[22]
Periodontal Therapy for Localized Severe Periodontitis in a Patient Receiving Fixed Orthodontic Treatment	Case Report	[23]
Interdisciplinary Treatment of a Patient with Severe Pathologic Tooth Migration Caused by Localized Aggressive Periodontitis	Case Report	[24]
Orthodontic Treatment After Induced Periodontal Regeneration in Deep Infrabony Defects	Case Series	[25]
Orthodontic Treatment of Patients with Severe (Stage IV) Periodontitis	Systematic Review	[26]

**Table 4:** Detailed assessment of the risk of bias across the 17 studies.

Study Title	Study Design	Tool Used	Domains Assessed	Overall Risk of Bias
Comparative evaluation of treatment of angular bone defects [27]	Controlled Clinical Trial	Newcastle-Ottawa Scale	Participant selection, outcome standardization, follow-up quality	Moderate
Evaluation of periodontal status after orthodontic treatment [28]	Pilot Study	Newcastle-Ottawa Scale	Selection bias, intervention consistency, missing data, small sample size	Moderate
Orthodontic movement into infrabony defects in advanced periodontal disease [29]	Prospective Observational	Newcastle-Ottawa Scale	Participant selection, outcome consistency, intervention protocol	Low to Moderate
Knowledge, attitudes, and interest in orthodontic treatment in adults with stage III–IV periodontitis [30]	Cross-Sectional Study	JBI Checklist	Participant selection, measurement validity, missing data, reporting bias	Moderate
Long-term stability of regenerative periodontal surgery and orthodontic tooth movement [31]	Retrospective Cohort	ROBINS-I	Confounding, selection bias, missing data, outcome measurement	Moderate to High
Orthodontic treatment simultaneous to periodontal therapy [32]	Randomized Controlled Clinical Trial	Cochrane Risk of Bias 2 (ROB 2)	Randomization, group comparability, clinical outcome measurements, selective reporting	Low to Moderate
Effect of orthodontic treatment on periodontally compromised patients [33]	Prospective Randomized Controlled Trial	Cochrane Risk of Bias 2 (ROB 2)	Randomization, clinical outcome assessment, selective reporting	Low
Orthodontic treatment in patients with aggressive periodontitis [34]	Before-After Clinical Study	Newcastle-Ottawa Scale	Selection bias, comparability of groups, exposure and outcome measurement	Moderate
Periodontal and orthodontic synergy in Stage IV periodontitis [35]	Retrospective Study	ROBINS-I	Confounding, selection bias, intervention classification, outcome reliability	Moderate
Synergy of regenerative periodontal surgery and orthodontics on QoL [36]	Multicenter Randomized Clinical Trial	Cochrane Risk of Bias 2 (ROB 2)	Randomization, blinding, outcome measurement, selective reporting	Low to Moderate
The effect of timing of orthodontic therapy on outcomes of regenerative periodontal surgery [37]	Multicenter Randomized Clinical Trial	Cochrane Risk of Bias 2 (ROB 2)	Randomization, blinding, outcome measurement, selective reporting	Low to Moderate
Periodontal parameters following orthodontic treatment in patients with aggressive periodontitis [38]	Before-After Clinical Trial	Newcastle-Ottawa Scale	Participant selection, intervention standardization, missing data, follow-up quality	Moderate
Orthodontic treatment of periodontally compromised teeth after periodontal regeneration [39]	Retrospective Study	ROBINS-I	Confounding, timing of interventions, outcome classification	Moderate
Immediate orthodontic treatment after periodontal regeneration of intrabony defects [40]	Long-term Retrospective Study	Newcastle-Ottawa Scale	Participant selection, follow-up quality, timing of interventions	Moderate
Regenerative periodontal surgery and orthodontic tooth movement in stage IV periodontitis [41]	Retrospective Cohort	ROBINS-I	Confounding, selection bias, missing data, classification of interventions, outcome measurement	Moderate to High
Periodontal regeneration and orthodontic treatment of compromised teeth [42]	Prospective Observational	Newcastle-Ottawa Scale	Confounding, follow-up quality, intervention timing	Low to Moderate
Malocclusions, pathologic tooth migration, and orthodontic treatment need [43]	Cross-Sectional Study	JBI Checklist	Participant selection, data accuracy, reporting bias, external validity	Moderate

**Table 5:** General characteristics of the 17 included studies, detailing study populations, sample sizes, demographic data, and classification according to the 2017 periodontal system.

Author/Year	Sample Size Calculation	Study Population	Patients	Sex (Female/Male)	Mean Age (±SD)	Age Range (Years)	2017 Classification Note
Ghouraba et al., 2024 [27]	Yes	Overerupted teeth with angular bone loss	10	Not specified	No data	30–55	Stage IV, Grade C
Jiao et al., 2019 [28]	Yes	Stage IV Grade C periodontitis	24	16/8	No data	18–35	Stage IV, Grade C periodontitis
Corrente et al., 2003 [29]	No	Advanced periodontal disease	10	8/2	No data	33–53	Stage IV, Grade C
Zasčiurinskienė et al., 2023 [30]	Yes	Stage III–IV periodontitis	96	67/29	45.7 (±10.2)	30–78	Stage III–IV, Grade C
Tietmann et al., 2023 [31]	Yes	Stage IV periodontitis	22	13/9	43.9 (±N/A)	29–62	Stage IV, Grade C, three patients with Grade B
Zasčiurinskienė et al., 2018 [32]	Yes	Periodontitis patients needing ortho	50	35/15	47 (±3.4)	25–55	Stage III, Grade B
Gehlot et al., 2023 [33]	Yes	Mild-moderate-severe periodontitis	36	23/13	47 (±3.0)	No data	Stage II, Grade B Stage III–IV, Grade C, as per CBCT findings)
Carvalho et al., 2017 [34]	Yes	Aggressive periodontitis and periodontally healthy subjects	20	17/3	25.0 (±5.22)	No data	Stage IV, Grade C and clinically healthy subjects
Garbo et al., 2022 [35]	No	Generalized Stage IV periodontitis	29	23/6	55.1 ± 6.5	44–68	Stage IV, Grade C
Jepsen et al., 2023 [36]	No	Stage IV periodontitis	43	26/17	45.4 ± 11.9	No data	Stage IV, Grade C
Jepsen et al., 2021 [37]	Yes	Severe periodontitis	43	26/17	45.4 ± 11.9	30–55	Stage IV, Grade C (6 patients Grade B)
Khorsand et al., 2013 [38]	No	Aggressive periodontitis	8	7/1	30 (±NA)	No data	Stage III/IV, Grade C
Tu et al., 2022 [39]	No	Stage III or IV and Grade B or C	21	11/10	40 (±NA))	23–66	Stage III or IV and Grade B or C
Dung et al., 2024 [40]	No	Patients presented with severe periodontitis and malocclusion	9	7/2	44.2 ± 9.0	30–59	Stage IV, Grade C periodontitis
Tietmann et al., 2021 [41]	No	Stage IV	48	60.4% females	45.3 (±NA)	29–66	Stage IV, Grade C
Roccuzzo et al., 2018 [42]	No	Severe periodontitis	48	28/20	44.3 ± 8.5	No data	Stage IV, Grade C (based on CAL ≥ 10 mm)
Zasčiurinskienė et al., 2023 [43]	Yes	Stage III–IV periodontitis, Grade A, B, and C	121	85/36	44 (±0.48)	30–78	Stage III–IV periodontitis

**Table 6:** Overview of study aims, populations, evaluation methods, results, and follow-up periods from selected clinical studies investigating the effects of orthodontic treatment on periodontally compromised patients.

Author/Year	Aim of Study	Study Group	Evaluation	Results	Follow-Up Period
Ghouraba et al., (2024) [27]	Compare GTR followed by OI versus OI followed by GTR in treating overerupted teeth with angular bone loss	10 patients (20 teeth) with overerupted teeth and angular bone loss	Clinical (PD, BOP, TM) and radiographic (CBCT for defect depth and bone area) evaluations	Group 1 (GTR + OI) showed significant short-term improvement; Group 2 (OI + GTR) showed better long-term results. G2 had less PD and TM at one-year follow-up.	One year
Jiao et al., (2019) [28]	Evaluate the effect of orthodontic treatment on periodontal status in patients with Stage IV/grade C periodontitis	24 patients with Stage IV/Grade C periodontitis	PD, BOP, RBH% before and after treatment	No significant changes in PD, BOP, or RBH% after treatment. Stable periodontal status was observed.	Not specified
Corrente et al., 2003 [29]	Evaluate the effects of combined periodontic and orthodontic therapy on periodontal tissue alterations following periodontal surgery and orthodontic intrusion in patients with advanced periodontal disease and pathologic flaring of frontal teeth	10 patients with advanced periodontal disease and extruded maxillary central incisors with infrabony defects	PD, CAL, radiographs (vertical and horizontal bone fill)	Significant reduction in PD (mean 4.35 mm), CAL gain (mean 5.50 mm), and radiological bone fill (vertical: 1.35 mm, horizontal: 1.40 mm). No root resorption or other adverse effects.	10 ± 2.6 months
Zasčiurinskienė et al., 2023 [30]	Determine interest in orthodontic treatment (OT) and its association with oral health status and knowledge about the disease in adults with Stage III–IV periodontitis	96 subjects with Stage III–IV periodontitis aged ≥ 30 years	Comprehensive periodontal-orthodontic examination, questionnaire (44 questions), and statistical analysis	56.3% expressed interest in OT. Stage IV periodontitis and Grade C were predictors for OT interest.	Not specified
Tietmann et al., 2023 [31]	Evaluate the long-term effectiveness of regenerative treatment with orthodontics	22 patients with Stage IV periodontitis, 256 intra-bony defects	Radiographic bone level (rBL), probing pocket depth (PPD)	Mean rBL gain: 4.48 mm after 10 years, 90% pocket closure, 4.5% tooth loss	10 years
Zasčiurinskienė et al., (2018) [32]	Compare the effect of orthodontic treatment timing on periodontal status in periodontally susceptible patients	50 periodontal patients, randomized into test and control groups	Clinical attachment level (CAL), probing depth (PD), gingival recession (REC)	Both groups showed CAL gain and PD reduction. The control group (periodontal therapy before orthodontics) had more PD sites of 4–6 mm healed compared to the test group.	6 years (2010–2016)
Gehlot et al., 2022 [33]	Evaluate the effect of fixed orthodontic treatment on periodontal health in periodontally compromised patients	36 adult patients with periodontitis (randomized into test and control groups)	Clinical (CAL, PD, PI, GI, BOP) and radiographic (ABL) assessments at baseline, start, and 1 year after ortho.	Significant improvement in periodontal parameters for both groups. No statistically significant difference between groups.	1 year
Carvalho et al., 2018 [34]	Evaluate the effects of orthodontic movement on periodontal tissues in patients with aggressive periodontitis (AP)	10 subjects with AP (ages 25.0 ± 5.22 years) and 10 healthy controls (ages 22.9 ± 5.23 years)	PPD, CAL, BoP, PI measured at baseline, after orthodontic treatment, and 4 months later	Significant improvements in all clinical parameters: PPD (−0.29 mm), CAL (+0.38 mm), BoP (−4%), PI (−11%)	4 months post-orthodontic treatment
Garbo et al., 2022 [35]	Evaluate the periodontal and orthodontic synergy in managing patients with Stage IV periodontitis and pathologic tooth migration	29 patients with Stage IV periodontitis and PTM	Clinical (CAL, PD, GI) and radiographic assessments	Significant CAL gain, reduced PD, improved esthetics, and functional outcomes	1 year
Jepsen et al., (2023) [36]	Evaluate the combined effect of regenerative periodontal surgery (RPS) and orthodontic treatment (OT) on periodontal parameters and quality of life in patients with Stage IV periodontitis and pathologic tooth migration over 24 months	43 patients with Stage IV periodontitis randomized into early orthodontic treatment (4 weeks post-RPS) and late orthodontic treatment (6 months post-RPS)	Clinical attachment level (CAL), probing pocket depth (PPD), and patient-reported outcomes (GOHAI index) at baseline, 6, 12, and 24 months	Early OT showed a statistically significant higher CAL gain (5.96 ± 2.1 mm) compared to late OT (4.65 ± 1.76 mm, *p* = 0.034). Pocket closure (PPD ≤ 4 mm) was achieved in 91% of defects with early OT and 90% with late OT. Quality of life significantly improved in both groups, with greater reductions in GOHAI scores for early OT.	24 months
Jepsen et al., 2021 [37]	Compare outcomes of early (4 weeks) vs. late (6 months) orthodontic therapy	43 patients with Stage IV periodontitis	CAL, PPD, BOP, pocket closure	No significant difference in CAL gain (5.4 mm for early vs. 4.5 mm for late OT). Pocket closure was 91% for early vs. 85% for late OT.	12 months
Khorsand et al., (2013) [38]	Evaluate periodontal parameters (probing depth, plaque index, defect width, and defect depth) following orthodontic treatment in patients with aggressive periodontitis	8 patients diagnosed with aggressive periodontitis (extruded maxillary incisors, infrabony defects, PPD ≥ 5 mm). Orthodontic treatment included intrusion and alignment after periodontal therapy.	Clinical parameters (PI, PPD, defect width, defect depth) were measured at baseline, 3 months, and 6 months.	Statistically significant reductions in PPD, PI, and defect dimensions at 3 and 6 months. Root length and papilla height remained stable.	6 months
Tu et al., 2022 [39]	Assess orthodontic treatment outcomes post-periodontal regeneration	21 patients with compromised teeth (Stage III or IV, Grade B or C)	PD, CAL, radiographic assessment	Improved periodontal stability, reduced PD and CAL	1 year
Dung et al., 2024 [40]	Evaluate the long-term impact of immediate orthodontic treatment after regenerative procedures for periodontal intrabony defects	9 patients with 17 intrabony defects	Probing depth, attachment level, bone fill	Mean PD reduction: 3.94 mm, CAL gain: 3.47 mm, Bone fill: 4.89 mm	12.8 years
Tietmann et al., 2021 [41]	Evaluate the outcomes of orthodontic tooth movement (OTM) following regenerative periodontal surgery in Stage IV periodontitis patients with pathologic tooth migration (PTM)	Patients with Stage IV periodontitis exhibiting PTM (*n* = 48).	Clinical (CAL, PD), radiographic (bone levels), and periodontal stability	Significant improvement in CAL and PD. Radiographic bone gain observed.	12 months
Roccuzzo et al., 2018 [42]	Investigate long-term outcomes of orthodontic treatment following periodontal regeneration	48 patients with severe periodontitis and pathologic tooth migration	Clinical measurements (PD, BOP, pus)	PD reduced from 6.3 ± 1.5 mm to 3.1 ± 0.6 mm; significant reduction in BOP and pus	10 years
Zasčiurinskienė et al., 2023 [43]	Assess the prevalence of malocclusions and orthodontic treatment needs in subjects with Stage III–IV periodontitis	121 subjects with stage III–IV periodontitis	Comprehensive periodontal–orthodontic examination	Class II malocclusion was most prevalent (49.6%), and PTM observed in 74.4% of maxillary AT. OTN needed in >50% subjects.	Not specified

## Data Availability

No new data were created or analyzed in this study.
